# Reproducibility of cervical range of motion in patients with neck pain

**DOI:** 10.1186/1471-2474-6-59

**Published:** 2005-12-13

**Authors:** Jan Lucas Hoving, Jan JM Pool, Henk van Mameren, Walter JLM Devillé, Willem JJ Assendelft, Henrica CW de Vet, Andrea F de Winter, Bart W Koes, Lex M Bouter

**Affiliations:** 1 Institute for Research in Extramural Medicine, VU University Medical Centre, Amsterdam, the Netherlands; 2 Department of Clinical Epidemiology, Cabrini Hospital and Monash University Department of Epidemiology and Preventive Medicine, Melbourne, Australia; 3 Department of Anatomy and Embryology, Faculty of Medicine, Maastricht University, the Netherlands; 4 NIVEL Foundation, Utrecht, The Netherlands; 5 Department of General Practice and Nursing Home Medicine, LUMC Medical Centre, Leiden, the Netherlands; 6 Department of Social Psychiatry, University of Groningen, the Netherlands; 7 Department of General Practice, Erasmus Medical Centre, Rotterdam, the Netherlands

## Abstract

**Background:**

Reproducibility measurements of the range of motion are an important prerequisite for the interpretation of study results. The aim of the study is to assess the intra-rater and inter-rater reproducibility of the measurement of active Range of Motion (ROM) in patients with neck pain using the Cybex Electronic Digital Inclinometer-320 (EDI-320).

**Methods:**

In an outpatient clinic in a primary care setting 32 patients with at least 2 weeks of pain and/or stiffness in the neck were randomly assessed, in a test- retest design with blinded raters using a standardized measurement protocol. Cervical flexion-extension, lateral flexion and rotation were assessed.

**Results:**

Reliability expressed by the Intraclass Correlation Coefficient (ICC) was 0.93 (lateral flexion) or higher for intra-rater reliability and 0.89 (lateral flexion) or higher for inter-rater reliability. The 95% limits of agreement for intra-rater agreement, expressing the range of the differences between two ratings were -2.5 ± 11.1° for flexion-extension, -0.1 ± 10.4° for lateral flexion and -5.9 ± 13.5° for rotation. For inter-rater agreement the limits of agreement were 3.3 ± 17.0° for flexion-extension, 0.5 ± 17.0° for lateral flexion and -1.3 ± 24.6° for rotation.

**Conclusion:**

In general, the intra-rater reproducibility and the inter-rater reproducibility were good. We recommend to compare the reproducibility and clinical applicability of the EDI-320 inclinometer with other cervical ROM measures in symptomatic patients.

## Background

Neck pain is a common musculoskeletal disorder. The point prevalence for neck pain in the general population of the Netherlands varies between 9% and 22% [1,2], and approximately one-third of all adults will experience neck pain during the course of 1 year [3]. Patients usually receive conservative treatment such as physical therapy or continued care by a General Practitioner (GP) [4]. A physical evaluation is often used for both the diagnosis and the evaluation of treatment success in patients with neck pain [5]. One aspect for the physical assessment of the cervical spine is the evaluation of active Range Of Motion (ROM). Active cervical ROM is difficult to measure because of compensatory movements, and it is influenced by aging and systemic disorders [6]. Several non-invasive methods for assessing the ROM have been available, such as visual estimation, two-arm goniometry, inclinometry, compass technology, video technology, electromagnetic technology and potentiometry. For the majority of these instruments the intra-rater and inter-rater reproducibility has not been tested adequately. Radiography has been proven to be of questionable reproducibility [6,7].

In an extensive critical appraisal of reliability studies on cervical ROM measures Jordan evaluated 21 papers for methodological rigor [8]. Commonly identified flaws in these reliability studies were low sample size, unclear selection criteria, the use of only healthy individuals, use of inadequate reliability statistics, the absence of a protocol, and questionable applicability in clinical practice.

In our experience the Cybex Electronic Digital Inclinometer-320 (EDI-320) is a practical tool for the objective measurement of active ROM [9]. One of the clinical advantages of the EDI-320 is that it does not have to be fitted on the patient and it is portable.

Previous studies using the EDI-320 have investigated the intra-rater and inter-rater reproducibility only in healthy subjects [10,11]. It is unknown whether these reproducibility results are applicable to patients with pain or stiffness in the neck. Consequently, the aim of our study is to determine the intra-rater and inter-rater reproducibility in patients with non-specific neck pain. We also assess whether the reproducibility can be improved when two ratings per rater are used instead of one rating. Furthermore, we evaluate whether the inter-rater reproducibility is affected by the severity of pain.

Results of reproducibility studies can be used for many purposes. One application is the determination of changes that can be detected beyond measurement error: the smallest detectable difference (SDD). In the present study we assess SDD for an individual patient.

## Methods

### Patient characteristics

Consecutive patients with neck pain, referred by local general practitioners for physical therapy in Zoetermeer, the Netherlands, were invited to participate. The selection criteria were: age between 18 and 70 years, pain and/or stiffness in the neck for at least 2 weeks, and written informed consent. Patients were excluded if they had received surgery in the cervical region or had evidence of specific pathology, such as malignancy, neurological disease, fracture, herniated disc or systemic rheumatic disease. Data on demographics (e.g. age and gender), clinical factors (duration, concomitant complaints), neck pain on a numerical 0–10 point scale ranging from 0 (no pain) to 10 (maximal pain), and disability assessed with the Neck Disability Index (NDI) [12] were collected by an independent research assistant prior to the actual active ROM measurements.

### Rater characteristics

The raters were two physical therapists with 3 months experience using the EDI-320 inclinometer (Lumex, Inc., Ronkonkoma, New York) [9] and both performed weekly cervical ROM assessments in another study. The measurement procedures were practiced on 5 healthy volunteers prior to the start of the present study.

### Measurement protocol

**Figure 1:**
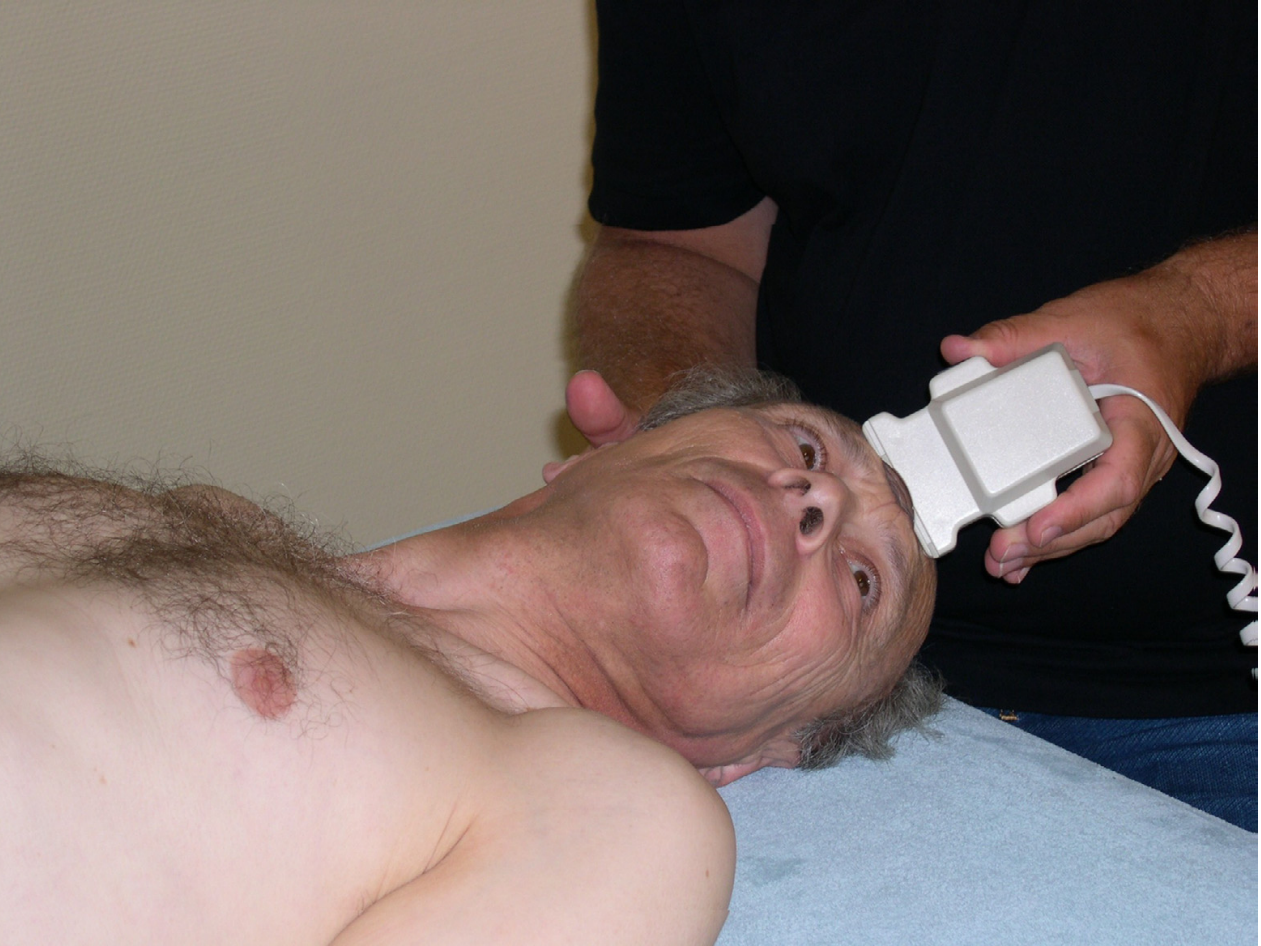
Picture of head position during rotation.

For the measurements of cervical flexion-extension and lateral flexion the patient was seated upright in a high chair, with the hands resting on the upper thigh. For the measurement of cervical flexion-extension, the position of 0 degrees was in maximal cervical flexion ("chin to chest"), followed by maximal cervical extension. Likewise, the measurements of lateral flexion were initiated with the position of 0 degrees in maximal lateral flexion to the left ("ear to left shoulder"), followed by maximal lateral flexion to the right. Because active ROM using the EDI-320 inclinometer can only be measured against gravity, the ratings of cervical rotation were performed with the patient in a supine position. The position of 0 degrees was in maximal left rotation, followed by maximal right rotation. During rotation the head slide over a cushioned treatment table and the patient was not allowed to make any compensatory lateral flexion with the head. See figure 1

We chose for full cycle ROM (for example: from left to right rotation) as the neutral head position is difficult to perform in half-cycle ROM (for example: from the neutral to left rotation) assessments in the cervical spine[7]. The reference point for the EDI was on the forehead for both flexion-extension and rotation, and right above the ear for lateral flexion. Throughout the motion, the physiotherapist maintained contact with the EDI and the reference point on the head.

The subjects were instructed to perform the movement and then to practice twice before performing the actual movement. The patient was instructed only to move the head, and to avoid compensatory movements in the thoracic or lumbar region. The patient was gently guided through the whole range of motion, and manual contact was applied by the rater. The patient was encouraged to perform a maximal movement until the end of the active ROM was reached, or until the pain prevented the patient from going any further.

### Procedure reproducibility study

Active ROM of the cervical spine was assessed twice in three planes in the following order: maximal flexion to maximal extension (2×), maximal lateral flexion from left to right (2×), and maximal rotation from left to right (2×). The time interval between measurement between the first and second ratings of a single rater was 5 minutes and the interval between raters was 10 minutes. The order of the raters was randomized using a computer generated random sequence table. At all times only one rater was present in the examination room, together with the research assistant. The research assistant recorded the number of degrees, which were electronically displayed on the EDI-320. In order to keep the raters blind for the outcome of measurement, the read out on the electronic display of the EDI-320 was concealed for both raters and patients. Thus, the raters were unaware of the previous measurements by the other rater.

### Data analysis

**Figure 2:**
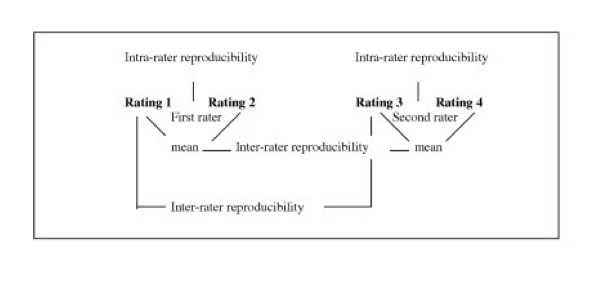
Flow diagram measurements and assessment of intra- and inter-rater reproducibility.

We used two different measures which are increasingly used in reproducibility studies: one measure to assess agreement and one measure to assess reliability [13,14]. Figure 2 shows an overview of the intra- rater and inter-rater comparisons we made.

### Agreement parameters

Parameters of agreement measure the ability to achieve the same value in two measurements, and gives an indication of the size of the measurement errors. We assess the 95% limits of agreement (LoA) according to Bland and Altman as a measure of agreement [15].

The mean difference between the scores of both raters was calculated, representing the systematic differences (bias) between the measurements. The standard deviation (SD) of this difference represented the extent to which the rater(s) recorded the same mean value in each plane. Then the 95% limits of agreement (LoA) were calculated (mean of the difference ± 1.96*SD), indicating the 'total error', systematic and random error together [16].

As no clear criteria exist for acceptable value of intra-rater and inter-rater agreements for active ROM outcome measures, we defined, a priori, that a difference in measurement between the raters of 10% of the total range of measurement values would be acceptable.

The Bland and Altman method can be visualized by plotting the differences between the first and the second ratings against the corresponding mean of the first and the second rating. This visual representation of agreement illustrates the magnitude and range of the differences, bias or outliers, and the relation between the magnitude of the differences and the magnitude of the mean values [15].

Based on the agreement results of rater A the smallest detectable difference (SDD) for an individual level was calculated for each movement by multiplying the SD of the differences by 1.96: 1.96* SD _change_. The SDD represents the change that can be detected by the EDI-320 beyond measurement error[17,18]

### Reliability parameters

Reliability parameters reflect the extent to which a measurement instrument can differentiate between patients[19]. If persons differ a lot, it is easier to distinguish them from each other, despite some measurement errors. In that case the measurement errors are related to the differences between the persons.

As a parameter of reliability the Intraclass Correlation Coefficient (ICC) was used (Figure 2) [16], Streiner and Norman 2003). We used ICCs which took systematic differences in the measurements into account. These ICCs are defined as the ratio of the variance among patients (patient variability) over the total variance (among patients, among raters plus the error variance), which ranges between 0 (no reliability) and 1 (perfect reliability). The cut-off point of ICC>0.75 was chosen a priori as an indication of acceptable reliability [20]. We used SPSS 9.0 statistical software (SPSS Inc., Chicago, Illinois) to calculate the ICCs [21]. In case the unit of analyses was the mean of two ratings by one rater, variances in which the raters were involved were divided by a factor 2 [21].

Figure 2 shows an overview of intra- and interrater comparisions

## Results

### Patient characteristics

During a period of 4 months (April 1999 – June 1999) 32 patients with neck pain were recruited. The mean age of patients included in this study was 45 years, and approximately 63% were female (Table 1). Patients had suffered from neck pain for a median duration of 13 weeks and in more than half of the patients the neck pain was recurrent. Patients rated the severity of their current neck pain, on average, as 4.2 on a numeric 11-point scale. The mean score for the NDI was 15.2 points (maximal disability: 50 points).

**Table 1:** Characteristics of patients.

Characteristics	Frequency* (n = 32)
Age (mean, sd)	45.5 (9.2)
Female	20 (62.5)
Headache	2 (6.3)
Trauma reported as cause	8 (25.0)
Reported stiffness of the neck	9 (28.1)
Previous neck pain episodes	18 (56.3)
Current pain 0–10 (mean, sd) †	4.2 (2.3)
NDI score (mean, sd) ‡	15.2 (8.3)
Duration neck pain (median; IQR^$^	13.5 (8.0, 25.5)

* Number of patients and % between brackets unless stated otherwise

† Current pain was measured on a numerical 11-point scale ranging from 0 = no pain to 10 = worst pain.

‡ Neck Disability Index: disability and pain measured by 10 items ranging from 0–5 points. Maximal disability 50 points

^$ ^IQR = Inter-Quartile Range

### Intra-rater agreement

**Figure 3:**
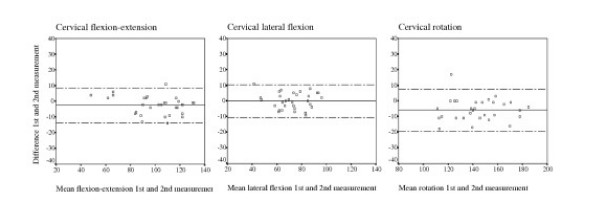
Plotted differences within rater A against the mean value of the first and second measurement for each patient for cervical flexion-extension, lateral flexion and rotation*. * Presented is the line of the mean difference (in the middle) and the limits of agreement (the two dotted outer lines corresponding with the +2 SD's or – 2 SD's of the mean difference between the 1^st ^and 2^nd ^rating by rater A)

The intra-rater agreement and reliability results are shown in Table 2 and Figure 3. Small but statistically significant systematic differences are seen for rotation for which the second rating of active ROM is higher, both for rater A and B (mean difference rater A: -5.9, 95% CI -8.4 to -3.4; rater B: -2.7, 95% CI -5.3 to -0.03) and for flexion-extension (rater A: -2.5, 95% CI -4.5 to -0.5). The limits of agreement were broadest for rotation. The standard deviation of the difference, representing the extent to which rater A achieved the same mean scores for the first and second rating, ranged between 5.3° (lateral flexion), 5.7° (flexion-extension) and 6.9° (rotation). From these, the limits of agreement were calculated. For any new patient it is expected with an approximate 95% probability that the difference between the two ratings of rater A should lie within the limits of agreement; which were -2.5 ± 11.1° for flexion-extension, -0.1 ± 10.4° for lateral flexion and -5.9 ± 13.5° for rotation. Figure 3 shows that the magnitude of the difference is not associated with their mean value, indicating that the mean difference and the standard deviation of the differences are adequate summary statistics of agreement. From the graphs, there were no indications of a larger variability for higher test values, i.e. homoscedasticity.

**Table 2:** Intrarater reproducibility analyses

Tested movements	*1st rating*		*2nd rating*		*1st-2nd rating*		LoA	ICC	95% CI
	mean	SD	mean	SD	mean	SD			
Rater A									
Flexion-extension	101.3	21.6	103.8	23.0	-2.5	5.7	-2.5 ± 11.1	0.96	0.93, 0.98
Lateral-flexion	72.6	13.8	72.7	14.4	-0.1	5.3	-0.1 ± 10.4	0.93	0.86, 0.97
Rotation	135.4	30.4	141.3	29.7	-5.9	6.9	-5.9 ± 13.5	0.96	0.91, 0.98
Rater B									
Flexion-extension	99.8	21.0	98.8	22.7	1.0	5.6	1.0 ± 11.1	0.97	0.93, 0.98
Lateral-flexion	71.8	12.3	72.4	14.1	-0.6	5.0	-0.6 ± 9.8	0.93	0.86, 0.96
Rotation	138.3	27.0	141.0	28.9	-2.7	7.4	-2.7 ± 14.4	0.96	0.92, 0.98

* LoA = 95% Limits of Agreement, ICC = Intra-class Correlation Coefficient, 95% CI = 95% Confidence Interval

### Inter-rater agreement

Regarding the inter-rater agreement, only minor systematic differences were observed between rater A and rater B for lateral flexion and rotation (Table 3). For flexion-extension there is a small but statistically significant systematic difference (mean 3.3; 95% CI 0.2 to 6.4). The standard deviation of the difference ranged between 8.7° (flexion-extension and lateral flexion) and 12.5° (rotation). The limits of agreement were 3.3 ± 17.0° for flexion-extension, 0.5 ± 17.0° for lateral flexion and -1.3 ± 24.6° for rotation.

**Table 3:** Interrater reproducibility analyses *

Tested movements	*Rater A*		*Rater B*		*Rater A-B*		LoA	ICC	95% CI
	mean	SD	mean	SD	mean	SD			
Mean of two ratings									
Flexion-extension	102.6	22.2	99.3	21.7	3.3	8.7	3.3 ± 17.0	0.95	0.90, 0.98
Lateral-flexion	72.6	13.9	72.1	13.0	0.5	8.7	0.5 ± 17.0	0.89	0.77, 0.94
Rotation	138.4	29.9	139.7	27.7	-1.3	12.5	-1.3 ± 24.6	0.95	0.90, 0.98
One rating									
Flexion-extension	101.3	21.6	99.8	21.0	1.6	10.1	1.6 ± 19.8	0.89	0.78, 0.94
Lateral-flexion	72.6	13.8	71.8	12.3	0.8	8.9	0.8 ± 17.5	0.77	0.58, 0.88
Rotation	135.4	30.4	138.3	27.0	-2.9	13.7	-2.9 ± 26.0	0.88	0.78, 0.94
Low pain intensity									
Flexion-extension	111.0	17.5	106.8	16.1	4.3	8.2	4.3 ± 16.1	0.92	0.79, 0.97
Lateral-flexion	77.0	11.3	76.0	11.6	1.1	8.9	1.1 ± 17.2	0.82	0.59, 0.93
Rotation	147.7	20.7	148.0	17.4	-0.3	12.8	-0.3 ± 25.1	0.88	0.71, 0.95
High pain intensity									
Flexion-extension	81.0	18.1	80.2	23.1	0.8	9.8	0.8 ± 19.2	0.95	0.76, 0.99
Lateral-flexion	61.4	14.1	62.3	11.5	-0.8	8.4	-0.8 ± 16.4	0.89	0.50, 0.98
Rotation	114.6	37.3	118.4	37.8	-3.8	12.1	-3.8 ± 23.8	0.97	0.89, 0.99

* LoA = 95% Limits of Agreement, ICC = Intra-class Correlation Coefficient, 95% CI = 95 % Confidence Interval

### Intra-rater and inter-rater reliability

The intra-rater reliability was high with ICCs ranging from 0.93 (lateral flexion rater A and B) to 0.97 (flexion-extension rater B). Likewise the inter-rater reliability was also good with ICCs of 0.89 or higher for all three planes.

### One rating versus two ratings per rater

Table 3 shows that when only one rating per rater was used instead of two, the limits of agreement were slightly wider and the ICC were slightly lower.

### The influence of pain on the inter-rater agreement and reliability

In addition, we compared patients with a high pain score (7 points or higher on a 0–10 point scale, n = 9) to patients with a low or moderate pain score (6 points or lower on a 0–10 point scale, n = 23). Patients with high pain intensity had lower active ROM values compared to patients with a low pain intensity (p ≤ 0.05). Although the standard deviations of the individual raters were higher in the high pain intensity group, the standard deviations of the mean differences were similar and consequently, the 95% limits of agreement did not differ much (Table 3). Therefore also the limits of agreement are similar. The ICC values in the high pain intensity group are slightly higher compared with those in the low pain intensity group.

### The smallest detectable difference

The mean active ROM values (mean of 4 ratings by 2 raters) were 100.9 degrees for flexion-extension, 72.4 degrees for lateral flexion and 139.0 degrees for rotation. The acceptable differences to be detected, defined as 10% of the used range of the scale, were therefore 10.1 for flexion-extension, 7.2 degrees for lateral flexion and 13.9 degrees for rotation

Based on the intra-rater agreement results (rater A), the SDD for an individual was 11.1 degrees for flexion-extension, 10.4 degrees for lateral flexion and 13.5 degrees for rotation. This means that only changes in cervical range of motion larger than these values can be detected beyond measurement error when a single physiotherapist performs both measurements. If the measurements on which the change in cervical range of motion is based are performed by two different raters than the SDDs were 17.0, 17.0 and 24.6 for flexion-extension, for lateral flexion and for rotation, respectively.

## Discussion

The first aim of this study was to investigate the intra-rater and inter-rater reproducibility of the assessment of range of motion in three planes for patients with neck pain, using the Cybex EDI-320 inclinometer. For intra-rater reproducibility we compared the first rating with the second rating of each rater and for the inter-rater reproducibility we compared rater A with rater B. Some systematic differences were observed, however these were small considering the overall active ROM in each plane for both the intra-rater and inter-rater agreement. Overall, we found good intra-rater and inter-rater reliability statistics (ICCs of 0.86 or higher). As expected both agreement and reliability were slightly higher for the intra-rater comparisons than for the inter-rater comparisons. High reliability does not necessarily mean that the raters agree in an absolute sense on the active ROM (agreement) [13,14]. For this reason we included both parameters of agreement and reliability in the present study.

The SDD, based on intra-rater agreement, for flexion-extension (11.1°) and rotation (13.5°) was almost equal to the cut-off values for our predefined criteria for an acceptable clinical difference (10.1° and 13.9°, respectively). However, for lateral flexion (10.4°) an acceptable clinical difference may be somewhat more difficult to detect as the SDD was higher than our predefined acceptable difference of 10% (7.2°).

Also measurements performed by different raters are insufficiently reproducible to detect the predefined difference of 10% of the used range of the measurement scale. However, this holds for SDDs calculated on the individual level. In research, when groups of patients are used the EDI-320 is sufficiently reproducible for all measurements of range of motion, because SDD values should be divided by √N to obtain SDD for group level, with a group size of N.

To minimize any random error, the inter-rater statistics were based on the mean of two ratings as outlined in our protocol. We investigated whether just one rating per rater instead of two would yield acceptable reproducibility statistics (second aim). Although a duplicate rating did not improve the reproducibility much, the 2nd rating with the EDI-320 can be done easily. Similarly, we evaluated whether reproducibility was affected by the severity of pain. Patients with high pain intensity had on average, less ROM compared to patients in the low pain intensity group (p ≤ 0.05). However, reliability and agreement were acceptable in both the group with low and high pain intensity.

We hypothesized that pain and limitation of movement could either increase or decrease during the course of a series of movements and thus pose sources of systematic variation to the assessment of reproducibility [22]. By comparing the first and second consecutive pair of ratings (independent of the rater), a statistically significant small, but not clinically relevant, difference was observed for flexion-extension (3.4 degrees difference: 95% CI 0.2 to 6.5). We therefore conclude that the effect of repeated movements on cervical ROM was minimal.

In the present study we looked at the intra-rater reproducibility by comparing two consecutive ratings with a minimal time interval and inter-rater reproducibility with an interval of approximately 10 minutes. The main reason for the choice of the time interval of 10 minutes was a practical one: we could measure a patient in one single visit. Our assumption was that within 10 minutes the patients will be stable on pain perception and range of motion. Had we chosen a larger time interval our results might have been different, however. Ideally, true intra-rater variability is evaluated for a disorder stable within the time frame evaluated. However, we consider a large time interval not desirable for the assessment of measurement variation because of the biological variation within subjects over time [6,23].

More than half of all studies on the reproducibility of cervical ROM have inappropriately used T-tests or repeated measures ANOVA, which are not considered true reliability statistics [8]. The ICC is used in only a few studies [8]. ICC values are known to be dependent on the variation in the study population [24]. As can be seen from the visual representation of agreement (Figure 2), the active ROM values for lateral flexion are somewhat more clustered together (a smaller range) than the other two planes. The more homogeneous values might give some explanation for the somewhat lower ICCs for lateral flexion, and the wider range of values result in higher ICCs for rotation. Likewise, the larger variation in active ROM values in the high pain intensity group might also explain the higher ICCs compared to the low pain intensity group.

Studies that measure ROM for patients with neck disorders are scarce. A systematic review identified that only 6 studies assessed reliability in patients with cervical disorders and of these only 2 studies had more than 30 subjects [8]. Two studies reported on the reproducibility of the EDI-320 for cervical ROM in healthy subjects [10,11]. The first one reported acceptable agreement results and found that more than 90% of the successive ratings for cervical flexion and lateral flexion by two raters were within a range between 0–10 degrees [10]. The other study only investigated flexion and extension, and reported moderate to high intra-rater reliability (flexion ICC 0.77, extension 0.79–83) and somewhat lower inter-rater reliability (flexion ICC 0.66–0.73; extension ICC 0.66–0.80) [11]. The authors of this study report that the reliability could be improved by using a standardized protocol. Comparison of ICC values between different studies is hampered by the dependency of ICC values on the variability of range of motion values of the population under study [14]. De Winter et al showed that for measurements of range of motion in 155 patients with shoulder complaints, the ICC were high for the affected shoulder (ICC = 0.83) and low for the non-affected shoulder (ICC = 0.28). This difference was completely due to variability of range of motion found for the affected shoulder, which was large and the non-affected shoulder, which was low.

The CROM device is the most frequently reported measure for cervical ROM and variable ICC values have been reported, both alone or when compared to other ROM instruments [8,25,26]. One study on patients with cervical spine disorders reported inter-rater ICCs for active ROM greater than 0.80 with the Cervical Range of Motion Device (CROM device) compared to ICCs lower than 0.80 for visual estimation and a universal goniometer (Youdas et al 1991). Considering the results of this study it would be interesting to directly compare the CROM device with the EDI-320 inclinometer in a future study.

Our population consisted of patients with non-specific neck pain, readers can compare the patient profile presented in this article with their own patients. The measurement procedure is quick and simple, which we hope will facilitate replication of our reproducibility design in other clinical settings.

## Conclusion

In general, the intra-rater reproducibility and the inter-rater reproducibility were acceptable, despite slight variations. We recommend that the reproducibility and clinical applicability of the EDI-320 inclinometer is compared with other cervical ROM measures in a symptomatic patient population.

## Pre-publication history

The pre-publication history for this paper can be accessed here:


